# Representation of research hypotheses

**DOI:** 10.1186/2041-1480-2-S2-S9

**Published:** 2011-05-17

**Authors:** Larisa N Soldatova, Andrey Rzhetsky

**Affiliations:** 1 Department of Computer Science, Penglais, Aberystwyth University, Wales, UK; 2 Department of Medicine & Department of Human Genetics, the University of Chicago, USA

## Abstract

**Background:**

Hypotheses are now being automatically produced on an industrial scale by computers in biology, *e.g.* the annotation of a genome is essentially a large set of hypotheses generated by sequence similarity programs; and robot scientists enable the full automation of a scientific investigation, including generation and testing of research hypotheses.

**Results:**

This paper proposes a logically defined way for recording automatically generated hypotheses in machine amenable way. The proposed formalism allows the description of complete hypotheses sets as specified input and output for scientific investigations. The formalism supports the decomposition of research hypotheses into more specialised hypotheses if that is required by an application. Hypotheses are represented in an operational way – it is possible to design an experiment to test them. The explicit formal description of research hypotheses promotes the explicit formal description of the results and conclusions of an investigation. The paper also proposes a framework for automated hypotheses generation. We demonstrate how the key components of the proposed framework are implemented in the Robot Scientist “Adam”.

**Conclusions:**

A formal representation of automatically generated research hypotheses can help to improve the way humans produce, record, and validate research hypotheses.

**Availability:**

http://www.aber.ac.uk/en/cs/research/cb/projects/robotscientist/results/

## Background

Research hypotheses are the heart of scientific endeavours; the accurate, unambiguous and operational representation of them is vital for the formal recording and analysis of investigations. Hypotheses should be represented and recorded so as to accurately capture the semantic meaning of the hypothesis and to promote the manual (or automated) design of experiments to test these hypotheses.

A number of projects aim to address the need to represent and record research hypotheses in a semantically defined form. Hypotheses in the Semantic Web Applications in Neuroscience (SWAN) Alzheimer knowledge-base are portions of natural language text which are represented as research statements (discourse-elements), and these are linked (via discourse-relations) to other discourse elements and citations which specify the author's name, article, journal, etc. [1]. Similarly, the Ontology for Biomedical Investigations (OBI) models hypotheses as the class *obi:hypothesis textual entity*, (here and further in the text we use *italic* for ontological classes and relations where appropriate), where hypotheses are part of *obi:objective specification* of *obi:investigation*[2,3]. The ART project [4] considers scientific papers as textual representation of scientific investigations, and uses the key classes from the generic ontology of experiments EXPO [5] to annotate papers. The class *expo:hypothesis* is used to annotate sentences which describe research hypotheses. For example, the paper b310850 from the ART Corpus of 225 annotated by experts papers [6] contains a sentence which has been annotated as a hypothesis:

<s sid="41"><annotationART atype="GSC" type="Hyp" conceptID="Hyp1" novelty="None" advantage="None">This means that whereas a central ligand may change chemical properties somewhat, this should only be a second order effect on the properties we are studying here.</annotationART></s>

The extraction of hypotheses from literature as textual entities, and the deposition of these hypotheses into publicly available, comprehensive, and semantically annotated collections opens up new prospects for knowledge sharing and exchange. The open and easy access to a whole range of alternative hypotheses reflecting a plurality of often contrarian theories, opinions, and views could significantly speed up the scientific progress. Unfortunately, it is typically hard to capture the precise semantic meaning of a hypothesis expressed as a textual entity; as sometimes it is impossible to understand the meaning and correctly process the hypothesis without reading a considerable portion of the surrounding text. Textual representation of the hypotheses retrieved from literature is mostly intended for “consumption” by humans, and has limited value for automatic processing.

A number of projects try to overcome this limitation and translate hypotheses into a machine-processable format. The HyBrow (Hypothesis Browser) tool for designing hypotheses, and evaluating them for consistency with existing knowledge, uses an ontology of hypotheses to represent hypotheses in machine understandable form as relations between objects (agents) and processes [7,8]. A hypothesis event is considered to be an abstract biological event. The ontology accommodates currently available literature data, extracted primarily from Yeast Proteome Database at a coarse level of resolution [9]. The Large-Scale Discovery of Scientific Hypotheses project aims to collect and make visible, comparable, and computable contrarian (with respect to a standard paradigm) hypotheses produced by the communities focusing on three classes of disease phenotypes (cancer, neuropsychiatric and infectious disorders) [10]. In this project hypotheses and supporting evidences are collected and structured in the form of statements, and then formalised as a propositional graph.

It is now likely that the majority of hypotheses in biology are computer generated. Computers are increasingly automating the process of hypothesis formation, for example: machine learning programs (based on induction) are used in chemistry to help design drugs; and in biology, genome annotation is essentially a vast process of (abductive) hypothesis formation. Such computer-generated hypotheses have been necessarily expressed in a computationally amenable way, but it is still not common practice to deposit them into a public database and make them available for processing by other applications.

In this paper, we extend the representation of hypotheses as textual entities to the representation of hypotheses, which are automatically generated by a machine, as logical entities following the HyBrow approach. This approach is also consistent with the representation of hypotheses by the Large-Scale Discovery of Scientific Hypotheses project. The proposed representation of research hypotheses is based on LABORS (the LABoratory Ontology for Robot Scientists) [11, Suppl.], and the representation of structural research units is based on LABORS and DDI (an ontology for the Description of Drug Discovery investigations) [12]). Instances of the hypotheses defined in LABORS, and instances of the recorded research units, are stored in a publicly available database [11,13]. All the hypotheses discussed below have been automatically generated by the Robot Scientist “Adam” (A Discovery Machine) [14]. An explicit semantically defined representation of hypotheses enables improvements in the representation of investigations designed to test those hypotheses, and in the consistency and validity checking of the conclusions about those hypotheses within the investigations.

The main contribution of this paper is a formal representation of research hypotheses in a logically defined form which enables scientists (robots or humans) to capture the precise semantic meaning of the hypothesis statements, and also promotes the design of experiment to test these hypotheses. The proposed formalism supports the decomposition of a generic hypothesis into specific hypotheses, and the representation of hypotheses as members of an exhaustive set of hypotheses covering a specific domain. The paper also proposes a framework for automated hypotheses generation, and the key components of the proposed framework are implemented for Adam. The authors discuss some of the limitations of the “conventional realism” in biomedicine for the formalisation of research hypotheses. An extension of the proposed approach, the probabilistic representation of research hypotheses, is also discussed. Our experiences in formally recording research hypotheses, and analysing automatically generated hypotheses are summarised as “lessons learned”. The examples in this paper are from the investigations run by Adam, the investigation into re-discovery of gene functions in aromatic amino acids pathway, and the investigation into novel biological knowledge, which are reported in [14] and [11]; all the information about the investigations, including procedures and data, is available at the Robot Scientist project web site [13].

## Methods

### Adam

**Figure 1:**
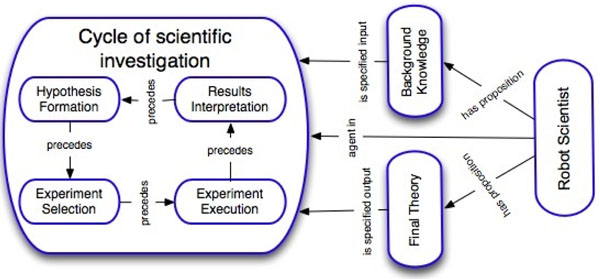
**A concept of a Robot Scientist.** A Robot Scientist is a physically implemented system which is capable of running cycles of scientific experimentation in a fully automatic manner: hypothesis formation, experiment selection, experiment execution, and results interpretation. The Robot Scientist system uses initial background knowledge and outputs new or updated knowledge.

We have developed the Robot Scientist “Adam” with the intended application domain of Systems Biology and Functional Genomics. The idea of a Robot Scientist is to combine laboratory automation, automated hypothesis formation, and other techniques from Artificial Intelligence to “close the loop” and automate the whole scientific process (see Figure 1) [11]. Adam has a -20°C freezer, 3 incubators, 2 readers, 3 liquid handlers, 3 robotic arms, 2 robot tracks, a centrifuge, a washer, an environmental control system, etc. It is capable of initiating ~1,000 new experiments and >200,000 observations per day in a continuous cycle. The representations proposed in this paper have been tested on Adam.

### LABORS

The proposed representation of research and negative hypotheses and its logical and textual representations are defined and tested within LABORS [11,15]. LABORS is designed to support investigations run by Adam for the area of Systems Biology and Functional Genomics. Thousands of experiments and corresponding hypotheses have been successfully recorded and re-used for further experimentation on the basis of LABORS. LABORS uses EXPO as an upper level ontology [5], and RO as a set of relations [16]. LABORS is expressed in W3C Ontology Web Language OWL-DL.

### DDI

The modelling of structural research units is based on both LABORS and DDI. DDI was designed to support investigations run by the Robot Scientist “Eve” for the area of drug discovery [12], and developed as an application of OBI. As a consequence, DDI uses BFO (Basic Formal Ontology) as an upper level ontology. Several compromise solutions were made within DDI in order to fit into the BFO framework. For example, the class *ddi:hypothesis* is defined as the subclass of the class *iao:information content entity.* DDI has been recently submitted to OBO, and negotiations about possible compromises to adjust for different representations are in progress.

### Datalog

Both LABORS and the corresponding database representations have been translated into Datalog in order to enable reasoning with the use of SWI-Prolog engine. The *is-a* and *instance-of* hierarchy has been translated into datalog with the use of one-ary predicates:

classA(subclassB).

classA(instance-ofC).

Other triplets have been translated into datalog with the use of bi-ary predicates:

Relation(classA,classB).

where a relation is a defined in LABORS or an additional predicate.

## Results

### Automatic generation of hypotheses

The Robot Scientist project is driven by the technological necessity to increase hypotheses production throughput (see Figure 1). Biological data are now produced at an industrial scale, while data analysis, and especially hypotheses formation, often remains manual. There is still strong belief that only human intelligence is capable of production of research hypotheses. The Robot Scientist project has proved that a machine can not only automatically generate scientifically valuable hypotheses, but also test them and make conclusions about their validity [14].

The nature of scientific discovery necessitates a succession of scientific theories: older dominant theories (paradigms) are contradicted by new experimental evidence, new paradigms are introduced, etc. [18]. The majority of discoveries in biomedicine are factual, e.g. gene G has function A, drug D can cure disease E, etc. The discovery of such scientific knowledge is based on abductive and deductive inferences, and modern technology is now able to automate the inference of possible new facts and their experimental confirmation [14]. The techniques for inductive inference are also in place, e.g. Inductive Logic Programming, but the results are still rather modest [17].

We argue that the automated formation of hypotheses requires the following key components:

1. Machine–computable representation of the domain knowledge.

2. Abductive or inductive inference of novel hypotheses.

3. An algorithm for the selection of hypotheses.

4. Deduction of the experimental consequences of hypotheses.

Adam has been designed to fully automate yeast growth experiments, and we show below how the key components of its hypothesis generation are implemented. The automated formation of hypotheses by Adam includes the following components:

**1. Yeast metabolic model** which encodes the background knowledge about yeast functional genomics domain. Our group has developed a logical formalism for modeling metabolic pathways (encoded in Prolog) [2]. This is essentially a directed graph with metabolites as nodes and enzymes as arcs. If a path can be found from cell inputs (metabolites in the growth medium) to all the cell outputs (essential compounds), then the cell can grow.

**2. Abductive Logic Programming for the inference** of missing arcs/labels in the yeast metabolic graph. Adam abductively hypothesizes new facts about yeast functional biology by inferring what is missing from a model. In our original work on robot scientists, we used a purely logical approach to hypothesis formation based on applying abductive logic programming to a logical model of a yeast metabolism subset [14]. Unfortunately, this general method is too inefficient to deal with large bioinformatic models. We therefore developed an alternative approach based on using standard bioinformatic methods – these are essentially based on abductive hypothesis formations [11]. Adam uses an automated strategy based on 1) finding the enzyme class (EC number) of the missing reaction, 2) finding genes that code for this EC class in other organisms, 3) finding homologous genes in yeast.

**3. The procedure for selection of hypotheses** which aims to satisfy the following combination of the selection criteria:

• it should encapsulate the maximum of information about a domain of interest;

• it should possess the maximum prior probability of being correct;

• it would require the minimum cost to test.

Adam investigates a finite hypothesis space, and uses a Bayesian approach that puts prior probabilities on the hypotheses. These priors have the potential to incorporate the complexity of the hypotheses [14].

**4. The deduction of experimental outputs**. Adam follows a hypothetico-deductive methodology. Adam abductively hypothesizes new facts about yeast functional biology, then it deduces the experimental consequences of these facts using its model of metabolism, which it then experimentally tests. To select experiments Adam takes into account the variable cost of experiments, and the different probabilities of hypotheses [14]. Adam chooses its experiments to minimise the expected cost of eliminating all but one hypothesis. This is in general a NP complete problem and Adam uses heuristics to find a solution. LABORS defines the class *labors:expected output* to model Adam's predictions for experiment results.

### Representation of automatically generated hypotheses

The class *labors:hypothesis* is defined as the subclass of the class *labors:proposition* which is equivalent to the class *iao:information content entity.* Whilst, LABORS has been designed to support automated investigations run by robots and therefore it does not have textual definitions, a sister DDI ontology for the Robot Scientist “Eve” provides a textual definition for the class hypothesis: “information content entity that is an assertion which is intended to be tested” [12]. The classes *labors:research hypothesis* and *labors:negative hypothesis* are defined as the subclasses of the class *labors:hypothesis.* The class *labors:hypothesis* is linked via the relation *has-representation* to the class *labors:representation* which has the subclasses *labors:textual representation* and *labors:logical representation.*

### Specification of hypotheses into different levels of granularity

**Figure 2:**
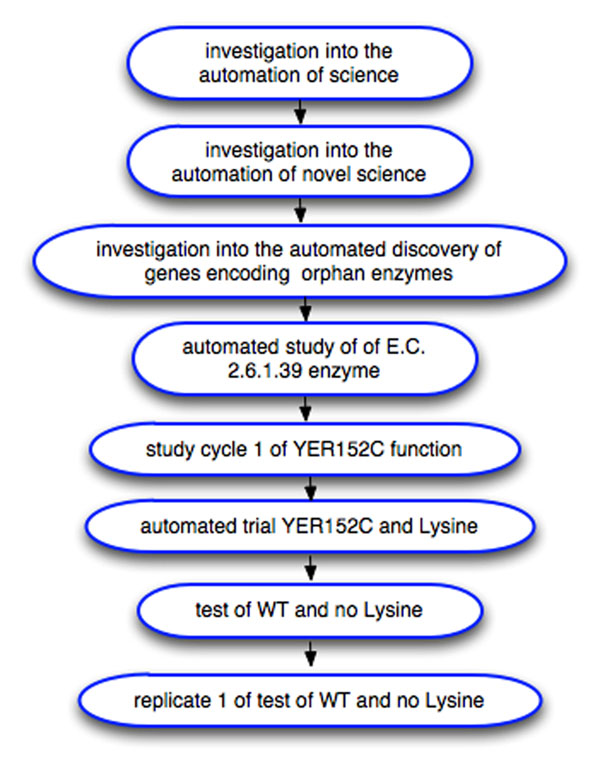
**Levels of investigations run by Adam.** An example of the levels of the representation of the investigation executed by the Robot Scientist “Adam” (a fragment). The relations are *has part*.

The automated investigations of robot scientist are generally complex, involving multiple study domains, and different levels of granularity. For example, the investigation into automation of science, in which Adam discovered novel knowledge about yeast genes, involves four different domains, and has 10 levels (see Figure 2) [11]. The levels are defined by a number of features including the corresponding hypotheses. On the top level is the hypothesis that it is possible to fully automate scientific discovery. This hypothesis is further decomposed into more specialised hypotheses, e.g. is it possible to automatically re-discover biological knowledge, that manual experiments would confirm the results obtained automatically by the robot, etc. At the lowest level of the investigation are hypotheses about quantitative yeast growth rates which are linked to the experimental data – optical density (OD) readings. The classes *labors:pregrowth optical data reading* and *labors:growth optical data* are modelled as subclasses of the class *labors:optical data reading* which is a subclass of the class *labors:experiment observation*. Complicated logical inferences are required in order to make the conclusion that scientific discovery can be fully automated from the basis of a large number of ODs (the inference procedures are available at the Robot Scientist project website [13]). The representation of hypotheses plays an essential role in the logical representation of such complex investigations. A machine can operate with hypotheses if and only if they are represented in a machine operable form.

LABORS enables the recording and storage in a relational database of the instances of the classes *labors:logical representation* which are linked to the instances of the classes *labors:research hypothesis* (H_0_) and *labors:negative hypothesis* (H_1_) [11]. The robot operates in a “closed world”, where a finite number of reactions, metabolites, and yeast strains are present. Therefore, the logical negation of hypotheses is possible. However, ontological representations utilise the “open world assumption” (OWA), where nothing outside of the ontologically defined collection of facts is known to be true or false. Relational databases operate under the “closed world assumption” (CWA), where everything outside the stated facts is false. In order to enable reasoning about the Adam's world over orthogonal data and knowledge representations, namely ontology, database, and models in Prolog, we chose to explicitly define negative hypotheses instead of inferring them.

**Figure 3:**
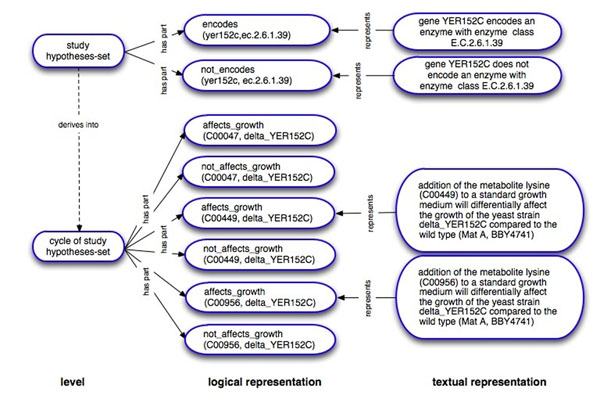
**Examples of hypotheses generated by Adam**. Each of the research and negative hypotheses from the hypotheses set of the study level is derived into more specialised hypotheses which are members of the hypotheses set of the cycle of study level. The hypotheses have logical and corresponding textual representations.

Let us consider the decomposition of hypotheses into more specialized ones in more detail (see Figure 3). Adam with the use of its background knowledge and bioinformatic facts, generates hypotheses about yeast genes and enzymes, i.e. gene YER152C encodes an enzyme with the enzyme class E.C.2.6.1.39 (the inference procedures are available at the Robot Scientist project website [13,20]) . The research hypotheses are encoded in the logical programming language Prolog, e.g.

encodes(yer152c,ec.2.6.1.39).

The enzyme class E. C. 2.6.1.39 is that of 2-aminoadipate transaminases. Adam also explicitly records the corresponding negative (null) hypotheses being tested:

not_encodes(yer152c,ec.2.6.1.39).

The research and negative hypotheses encoded in Prolog are stored in a relational database as instances of the class *labors:logical representation*. A logical representation of hypotheses is used to communicate with modules of Adam's software. For the convenience of humans, research hypotheses can be also translated into natural language text, i.e.:

gene YER152C encodes an enzyme with enzyme class E.C.2.6.1.39.

This is defined in LABORS as an instance of the class *labors:textual representation*.

Adam used abduction to form hypotheses. A real physical experiment is generally required to confirm (or to increase the probability) that a hypothesis is correct. However, such entities as the gene YER152C and an enzyme with the enzyme class E.C.2.6.1.39 exist only in Adam's memory, and not in Adam's physical world. In the real physical world Adam can operate only with yeast strains and metabolites. The hypothesis that gene YER152C encodes an enzyme with the enzyme class E.C.2.6.1.39 therefore has to be specialized to such a level that the robot can physically test the hypothesis. Using its background knowledge, Adam infers that if the research hypothesis is correct, then the addition of the following metabolites with the KEGG numbers C00047, C00449, and C00956, correspondingly, to growth medium for a yeast strain with a removed gene YER152C would restore the yeast growth rate (see Figure 3 and [13] for the inference procedures):

affects_growth(c00047,delta YER152C).

affects_growth(c00449,delta YER152C).

affects_growth(c00956,delta YER152C).

An example of the text representation of these new hypotheses is:

addition of the metabolite lysine (C00449) to a standard growth medium will differentially affect the growth of the yeast strain delta_YER152C compared to the wild type (Mat A, BBY4741).

If the metabolites are available, then using the yeast strain YER152C from its yeast strains library, Adam can physically test the hypotheses above. Adam designs microtitre plate layouts with controls and replicates in order to collect enough statistics to accurately analyse the results and runs the experiments. The class *labors:plate layout* is defined as the subclass of the class *labors:design*. In a series of experiments Adam tries to decide whether the difference in growth rate of two strains is significantly different and whether this difference can be attributed to differences in experimental conditions. In each case Adam compares four experimental setups: (1) a yeast strain with specific gene deleted and growing on a defined medium, (2) the same strain growing on the defined medium with a metabolite added, (3) wild type (WT) strain growing on the defined medium, and (4) WT strain growing on the defined medium and the metabolite. These experimental setups are combined within *labors:trial*. To make this decision, Adam uses decision trees and random forests combined with re-sampling methods. The deletion strains are mutant versions with genes removed that hypothesized to encode an orphan enzyme. Adam uses standard 96-well plates to grow the yeast, which enabled 24 repeats of each strain and medium combination. To control for intra-plate environmental effects, Adam uses *labors:latin squares strategy* of experiment design which is defined as the subclass of the class *labors:normalization strategy*.

**Figure 4:**
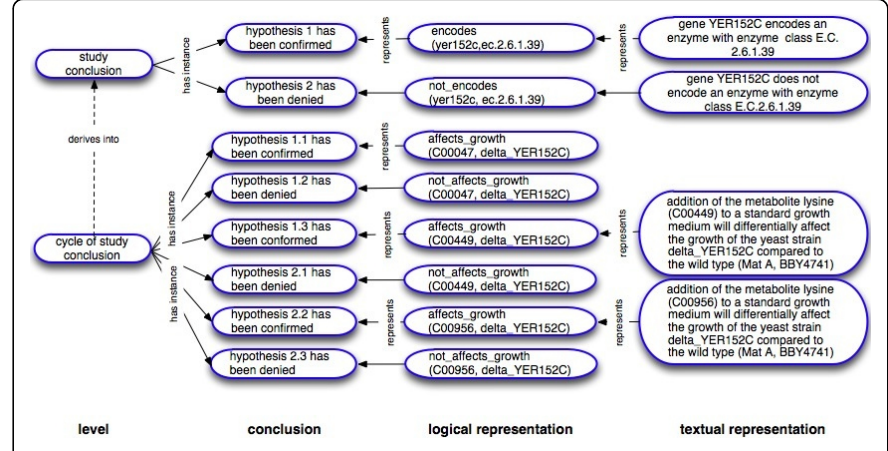
**Examples of results and conclusions produced by Adam**. Each of the research and negative hypotheses from the hypotheses set of the hypotheses set of the cycle of study level has been tested, observations analysed, decision procedures invoked and conclusions have been made. The results are expressed with the use of the same terms as the corresponding hypotheses. The results have logical and corresponding textual representations. Conclusions are made on the basis of the results with the use of decision procedures.

The results of the study are represented with the use of the same terms that were employed to encode the hypotheses (see Figure 4):

affects_growth(c00047,delta YER152C).

not_affects_growth(c00449,delta YER152C).

The corresponding textual representation of the result is:

addition of the metabolite lysine (C00449) to a standard growth medium differentially does not affect the growth of the yeast strain delta_YER152C compared to the wild type (Mat A, BBY4741).

The corresponding *labors:conclusion* or interpretations of the experiment results are expressed in the following form:

hypothesis X has been confirmed.

or

hypothesis X has been denied.

The conclusions are made following the corresponding decision procedures on the basis of the results (see the procedures at [13]). A more generic conclusion is made on the basis of more specific conclusions that correspond to more specific hypotheses. For example, a conclusion that a generic hypothesis is confirmed may be made if two out of three more specific hypotheses have been confirmed.

LABORS supports explicit and unambiguous recording of not only observations (i.e. ODs) (which is commonly done), but also the experimental results (i.e. predicate(metabolite,yeast_strain)), the corresponding conclusions (i.e. hypothesis X has been confirmed), and decision procedures employed for making those conclusions. The classes *labors:experiment observation*, *labors:result*, *labors:conclusion* are subclasses of the class *labors:research outcome*.

If hypotheses and conclusions of an investigation are recorded in this way, then it is possible to check how exactly each conclusion has been made: on what basis, and following what assumptions. If everything is explicitly recorded, then it is objectively possible to check which procedures were used, if the conclusions are valid, if they correspond to the stated hypotheses or those hypotheses have been replaced by related but different ones, etc. We argue that in the future all scientific investigations will be (or, at least, should be) recorded and reported in a similar way to enable complete consistency and validity checking of the results - these checks could potentially be done automatically.

### Sets of hypotheses for cyclic investigations

Robots can potentially generate thousands of hypotheses and test them in parallel. However, even for robots it is generally not practical to exhaustively test all possible hypotheses as hypothesis spaces can be very large. Adam selects hypotheses and designs experiments to test them following the combination of the selection criteria described in the previous sections. Such selected hypotheses are not completely independent, and LABORS models them as the class *labors:hypotheses set* (the subclass of the class *labors:collection*) where each particular hypothesis is a member of the set. A set of hypotheses is tested in cycles. Each cycle of investigation has a specified input *labors:hypotheses set*. Adam designs and runs experiments to test each hypothesis from the set. Adam then analyses the results of the experiments, and makes conclusions about whether a particular hypothesis has been confirmed or rejected. The rejected hypotheses are eliminated from the input *labors:hypotheses set* and the remaining set of hypotheses are considered as a specified output of the current *labors:cycle of study*. Adam updates its current model of metabolism and generates a new set of hypotheses, where the rejected on previous cycles hypotheses are excluded. This *labors:hypotheses set* is considered as a specified input for the next *labors:cycle of study*. Adam continues to run cycles of studies until the *labors:hypotheses set* contains only one hypothesis or the robot runs out of resources [14]. In the event that all hypotheses are eliminated a backtracking procedure is invoked [11]. If all hypotheses are eliminated, then the correct hypothesis, which is known *a priori* to be in the set, has been rejected. This can occur because Adam's observations are noisy. In such a case a backtracking procedure does more experiments to try to decide which hypothesis has been wrongly eliminated.

The analysis of the research hypotheses which were produced within Adam's investigations enabled us to improve the logical representation of the structural units of general scientific investigations by introducing new research units: *trial*, *study*, *cycle of study*, and *replicate* (see the next section and [12] for more detail)*.*

## Discussion

### Restrictions in the ontological representation of scientific discourse elements

#### OBI limitations

Currently prevalent ontological representations are not sufficient for the recording of hypotheses sets and complex (particularly cyclic) investigations. The most advanced project with the aim to support formal description of scientific investigations is OBI [3,21]. OBI aims to support the detailed description of investigations from the whole area of biomedicine. OBI descriptors include all phases of the investigation process, such as planning, execution and reporting, information and material entities that participate in these processes, as well as roles and functions. OBI intends to serve as the standard for the recording of biomedical investigations.

OBI represents a state-of-the-art for a cross-disciplinary formalisation of biomedical investigations, but it has its limitations. OBI defines investigations and study design executions in such a way that they cannot have inputs. For example, hypotheses formed in *obi:hypothesis generating investigation* (an investigation in which data is generated and analysed with the purpose of generating new hypothesis) cannot be passed to *obi:hypothesis driven investigation* (an investigation with the goal to test one or more hypothesis) (see also the classes *expo:hypothesis forming investigation* and *expo:hypothesis generating investigation* which have been introduced before OBI [5]).

To overcome these difficulties both LABORS and DDI in addition to the class *obi:investigation* define a number of structural research units: *study, cycle of study, trial,* and *replicate*, mainly according to the hypotheses tested within the research unit. For example, *replicates* test identical hypotheses, and have identical study designs; and *cycles of study* test hypotheses sets in cycles (for more detail and definitions of the research units see [12]).

OBI aims to represent the most typical investigations in biomedicine. Biomedical investigations are often complicated, but they are rarely as complex as the automated investigations run by robot scientists. Therefore, we do not propose that the OBI Consortium has to define or import more structural research units in order to support the representation of automated investigations - although in the near future it may become a necessity. We instead suggest that the definition for the key class *obi:investigation* should be changed in such a way so biomedical investigations can have research hypotheses as specified inputs.

#### BFO limitations

The ontological representations of biomedical research have more serious limitations than those discussed in the previous section. The concern is how suitable they are for the representation of theories, models, and research hypotheses – essential components of science [22]. Contemporary biology is complex, multidisciplinary and information-rich science. It necessarily produces diverse and often competing theories and conclusions, alternative hypotheses, data conceptualizations and interpretations. Ontologies as formal representations of knowledge should enable common understanding of key elements of biological knowledge and support knowledge sharing and exchange.

Open Biomedical Ontologies (OBO) are designed to support annotation of biological data and results, multidisciplinary cross-domain queries, management and exchange of biomedical knowledge [23]. Members of the OBO Foundry are committed to using the same designing principles in order to ensure their interoperability and orthogonality. OBO Foundry recommends using BFO as an upper ontology to ensure that OBO-ontologies are compatibly organised [24]. The advantage of such an approach is that ontologies can be developed and curated in parallel without duplication of efforts, and that OBO-ontologies can be combined if applications require it.

However, due to their adherence to BFO, the OBO-ontologies are limited to only classes with instances in the real world. BFO does not allow the inclusion of universals (entities which can be instantiated in many things) that have no instances in the reality into BFO-based ontologies, and considers them to be outside of the realistic approach [16,19,22]. Thus, from the BFO point of view, unconfirmed theories, models, hypotheses do not exist. Yet, biologists need to communicate such key components of their research, and OBO-ontologies in the present state are straggling to support this requirement. The definition of hypotheses as textual entities (like in OBI, IAO) is a clever compromise between the biologists' needs for unconfirmed entities and BFO. Instances of textual entities do exist in reality, e.g. in printed texts. However, this is only a partial solution, and one which arguably masks the central problem; and it is ill suited for applications outside of text mining. For example, the hypothesis

encodes(yer152c,ec.2.6.1.39).

at the time when Adam produced it has no instances in the reality. It exists only in the robot's memory as a number of charged transistors, and has no any associated textual entities. Only when the decision to select this hypothesis for the inclusion into a hypotheses set is made, and it is recorded in the database as a logical entity and also can be communicated to other programs and possibly to humans, does it exist as a textual entity. More importantly, it is unknown if the hypothesis statement is true or not. In fact, further experiments have confirmed that the statement is true with a certain degree of confidence. However, at a time of its confirmation the statement is no longer considered as a hypothesis, but as a result or a confirmed fact.

In general, in science there is no absolute confirmation: each hypothesis or theory with significant generality of claims is supported by evidences with a certain degree of confidence, and never reaching absolute certainty (while approaching it in some instances).

Another problem is how to include into a scientific ontology alternative and even contrary hypotheses and keep the ontology logically consistent. Researchers need a way to formalise various, sometimes contradictory, scientific discourse elements, e.g. different views, opinions, believes, and be able to reason over them. To support such needs, OBO Foundry might consider adopting a wider view on what exists.

### Towards a hypothesis ontology: probabilistic reasoning

In their eloquent book, Howson and Urbach [25] argue that Bayesian inference provides the only logically consistent way of reasoning about scientific hypotheses. Competing hypotheses should be compared with each other in terms of their posterior probability on given evidence (data). When a hypothesis is formulated with the aid of probability calculus as a generative model (that is, it describes how evidence is generated stochastically according to the hypothesis), we can explicitly compute the probability of evidence. This probability is commonly computed when research requires estimating model parameters given particular scientific hypothesis. However, scientists implicitly use different prior probabilities for competing hypotheses. Any competitive scientific hypothesis must provide a positive probability of generating the already existing evidence (or it should be rejected). When the amount of evidence is moderate, prior probability of hypothesis can affect results in a profound way. Therefore, we suggest that ontological descriptions of hypotheses should explicitly address probabilistic relations between hypotheses and evidence, and the multiplicity of prior distributions over hypotheses.

Specifically, we should be able to represent prior probabilities associated with competing hypotheses. Obviously, for alternative or disjoint hypotheses (competing to explain the same evidence), these probabilities should not exceed 1 when summed. We would need to represent multiple sets of prior probabilities (associated with different experts) for the same set of hypotheses. We should be able to specify support of a given hypothesis with regard to specific evidence as a posterior probability of a research hypothesis given the dataset. The hypothesis ontology should also allow the description of relations among hypotheses (e.g., are two hypotheses compatible or mutually exclusive?).

Clearly, different scientists within the same community can weight the same set of hypotheses in very different ways. Humans are notoriously bad at estimating the uncertainty of probabilities. Therefore, we suggest that ontological descriptions of hypotheses should explicitly record how prior probabilities have been obtained and what their uncertainties are.

Finally, we should be able to represent expert–hypothesis–evidence relations (expert-hypothesis-dataset–specific posterior probabilities). We believe that ontological modelling of this type is essential for large-scale automation of scientific reasoning.

## Conclusions

Here we summarise what lessons were learned from the representation of the automatically generated hypotheses by robots and how this might be useful for the improvement of the formulating and recording of research hypotheses produced by humans.

**Explicitness.** Research hypotheses should be expressed and recorded explicitly, unambiguously, and completely, so the semantic meaning of the hypothesis statement can be captured without additional information. (It is still common in the reporting of science for research hypotheses stated in the introduction to be implicitly replaced by other hypotheses in the conclusions [5]). It is also important to explicitly record hypotheses formed during investigations so that other researchers can easily find them (e.g. using text mining) and test them. This would speed up scientific progress.

**Operational approach.** Researchers should aim to formulate hypotheses in operational ways, so it is clear from hypotheses statements how to design experiments to test them. Hypothesis statements should contain only well defined entities and relations between them.

**Systematic approach.** The automated approach for hypotheses generation has an advantage of being systematic. All possible hypotheses for a study domain are considered, and the best are selected for testing. The concept of “the best hypothesis” is explicitly defined, i.e. as the most probable, cheapest, most informative one. Such a systematic approach should be adopted by humans for the assessment of research hypotheses.

**Statistical significance and reliability**. Researchers often report results that have been obtained without a sufficient number of experimental replicates, and therefore with unknown reliability. Adam executes 24 replicates of each study. This allows Adam to detect statistically significant differences in the yeast growth that are often missed by human-investigators [11]. This demonstrates the importance of the collecting the experimental data over a large enough number of repeated experiments to ensure statistical significance and reliable reproducibility of the results.

**Learning from negative results.** The hypotheses that have been rejected provide information about the domain of study. Therefore it is important to record and store the rejected hypotheses. Unfortunately, it is not a normal scientific practice to report negative results.

## Authors' contributions

RDK has conceived and implemented the idea of automated hypotheses generation by robot scientists. LNS has suggested the hierarchical representation of hypotheses and hypotheses sets. LNS drafted the manuscript. AR has analysed the representation of alternative and contrarian hypotheses and theories, and drafted the discussion section. All authors read and approved the final manuscript.

## Competing interests

The authors declare that they have no competing interests.
